# The Bile Acid TUDCA Improves Beta-Cell Mass and Reduces Insulin Degradation in Mice With Early-Stage of Type-1 Diabetes

**DOI:** 10.3389/fphys.2019.00561

**Published:** 2019-05-15

**Authors:** Gabriela Alves Bronczek, Jean Franciesco Vettorazzi, Gabriela Moreira Soares, Mirian Ayumi Kurauti, Cristiane Santos, Maressa Fernandes Bonfim, Everardo Magalhães Carneiro, Sandra Lucinei Balbo, Antonio Carlos Boschero, José Maria Costa Júnior

**Affiliations:** ^1^Laboratory of Endocrine Physiology and Metabolism, Biological Sciences and Health Center, Western Paraná State University (UNIOESTE), Cascavel, Brazil; ^2^Obesity and Comorbidities Research Center, Institute of Biology, University of Campinas (UNICAMP), Campinas, Brazil

**Keywords:** type 1 diabetes, insulin, glycemic control, bile acid, TUDCA

## Abstract

Type 1 diabetes (T1D) is characterized by impairment in beta-cell mass and insulin levels, resulting in hyperglycemia and diabetic complications. Since diagnosis, appropriate control of glycaemia in T1D requires insulin administration, which can result in side effects, such as hypoglycemia. In this sense, some bile acids have emerged as new therapeutic targets to treat T1D and T2D, as well as metabolic diseases. The taurine conjugated bile acid, tauroursodeoxycholic (TUDCA) reduces the incidence of T1D development and improves glucose homeostasis in obese and T2D mice. However, its effects in early-stage of T1D have not been well explored. Therefore, we have assessed the effects of TUDCA on the glycemic control of mice with early-stage T1D. To achieve this, C57BL/6 mice received intraperitoneal administration of streptozotocin (STZ, 40 mg/kg) for 5 days. Once diabetes was confirmed in the STZ mice, they received TUDCA treatment (300 mg/kg) or phosphate buffered saline (PBS) for 24 days. After 15 days of treatment, the STZ+TUDCA mice showed a 43% reduction in blood glucose, compared with the STZ group. This reduction was likely due to an increase in insulinemia. This increase in insulinemia may be explained, at least in part, by a reduction in hepatic IDE activity and, consequently, reduction on insulin clearance, as well as an increase in beta-cell mass and a higher beta-cell number per islet. Also, the groups did not present any alterations in insulin sensitivity. All together, these effects contributed to the improvement of glucose metabolism in T1D mice, pointing TUDCA as a potential therapeutic agent for the glycemic control in early-stage of T1D.

## Introduction

Type 1 diabetes is an autoimmune disease with progressive destruction of pancreatic beta-cells (Atkinson et al., 2014; Maganti et al., 2014) triggered by a genetic predisposition combined with environmental factors, resulting in a deficiency in insulin production (Cnop et al., 2005; Daneman, 2006). Besides, an increase in the insulin clearance also seems to contribute to the reduction of plasma insulin levels, observed in T1D rodents (Philippe et al., 1981). Inadequate management of the disease results in hyperglycemia, which can lead to various complications, such as neuropathy, retinopathy, nephropathy (Nathan et al., 2005; Lind et al., 2009), and cardiovascular disease (Lind et al., 2014). Because insulin plays a pivotal role in glycemic control, exogenous administration of insulin is required in early and advanced stages of T1D (Chiang et al., 2014; Henriksson et al., 2016; Katsarou et al., 2017).

Despite the availability of exogenous insulin, proper control of blood glucose in T1D patients is difficult to maintain (O’Hara et al., 2017). Insulin therapy is usually associated with some side effects, such as hypoglycemia and weight gain (Dewitt and Hirsch, 2003; Bergenstal et al., 2010), which is aggravated by the lack of patient compliance with adequate treatment, since the condition is more common in children and adolescents (Hilliard et al., 2013; Hynes et al., 2016, 2017). Thus, molecules that could be used for glycemic control, without side effects or compliance concerns, are a potential treatment for T1D.

**FIGURE 1:**
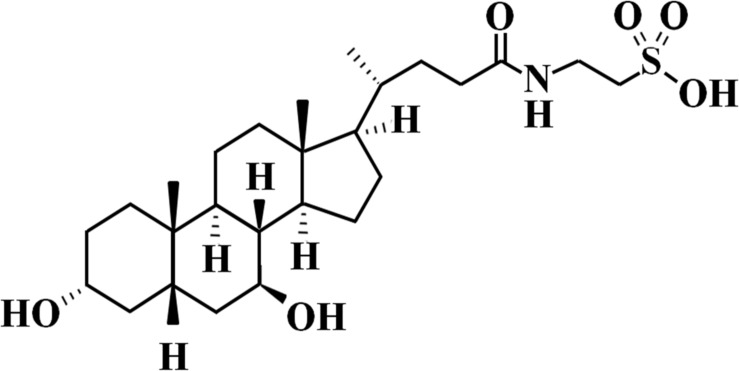
Tauroursodeoxycholic Acid (TUDCA) chemical structure. Molecular Formula: C_24_H_45_NO_6_S.

In this context, the bile acid TUDCA (Figure 1) has emerged as a possible candidate for T1D treatment due to its beneficial effects on glucose homeostasis (Da-Silva et al., 2011). TUDCA is synthesized in hepatocytes from the conjugation of UDCA with the amino acid taurine (Hagey et al., 1993). In the liver, TUDCA stimulates the sphingosine-1-phosphate receptor 2 (S1PR2), which activates insulin signaling through the activation of the PI3K/Akt pathway (Studer et al., 2012; Chiang, 2013). The S1PR2 activation by TUDCA also increases IDE expression in the liver, improving insulin clearance in an insulin resistance mice model (Vettorazzi et al., 2017). In addition, TUDCA acts as a chemical chaperone, reducing endoplasmic reticulum stress in hepatocytes (Ozcan et al., 2006) and pancreatic islets (Lee et al., 2010). In the pre-diabetic stage, TUDCA treatment reduces T1D incidence, improving beta-cell survival and preservation of islet architecture (Engin et al., 2013). Therefore, the effects of TUDCA on pancreatic islet and beta-cell mass, as well as its effect on insulin clearance and IDE modulation in the liver, after T1D induction, remain unclear. Using this technique, we tested the possible effects of TUDCA in reverting hyperglycemia in streptozotocin-induced early-stage T1D mice, focusing on pancreatic islet and liver regulation.

## Materials and Methods

### Reagents

Tauroursodeoxycholic acid was purchased from Calbiochem (São Paulo, Brazil, cat. 580549), and the insulin kit was acquired from Millipore (Darmstadt, Germany, cat. #EZRMI-12K). Streptozotocin was purchased from Sigma-Aldrich (St. Louis, MO, United States, S0130-1G). Western Blot reagents were purchased from Bio-Rad (Madrid, Spain). IDE antibody was acquired from Abcam (Cambridge, United Kingdom), and Tubulin was acquired from Sigma Aldrich (St. Louis, MO, United States). The insulin antibody was acquired from Dako North America (Carpinteria, CA, United States, ref. A0564). The remaining reagents were purchased from Sigma Aldrich.

### Animals

All experiments involving animals were approved by the Animal Care Committee at UNICAMP (License Number: 4747-1). Male, 8-week-old C57Bl/6 mice were obtained from the breeding colony at UNICAMP and maintained at 22 ± 1°C on a 12 h light-dark cycle. As in the Furman (2015) protocol to induce early stage of type 1 diabetes in the experimental group (STZ *n* = 21), an intraperitoneal (i.p.) injection of STZ (40 mg/kg, STZ was dissolved in 0.5 M citrate buffer, pH 4.5) was administered for 5 days. The same volume of a citrate buffer was injected in the control group (CON *n* = 6). Animals with fasting blood glucose levels above ≥ 200 mg/dL 12 days after the STZ injection were considered diabetic.

### Experimental Protocol

Once diabetes was confirmed in the STZ mice (fast glycemia > 200 mg/dL), the control group was excluded from other experiments, and was used only for insulin tolerance test, measurement of insulin levels and beta-cell mass in order to validate our early stage T1D model (Supplementary Figures 5–7). Diabetic mice were randomly selected and divided into the 2 following groups: (1) the STZ group (*n* = 10) received PBS intraperitoneally (i.p.), and (2) the STZ+TUDCA group (*n* = 11) received 300 mg/kg TUDCA (dissolved in PBS) i.p. The injections were administered for 24 days. The fasting glucose and body weight of both groups were measured on the 7th and 15th day of the treatment. Mice were killed on the 24th day of treatment in a CO_2_ chamber and decapitated for blood collection. The pancreas and liver were removed for posterior analyses.

### Intraperitoneal Glucose (ipGTT) and Insulin (ipITT) Tolerance Tests

On the 20th day of TUDCA treatment, the mice were subjected to 12 h fasting to perform the ipGTT. The fasting blood glucose level was measured (time 0) by a glucometer. After, the mice received an i.p. glucose dose of 1 g/kg body weight, and glycemia was measured at 15, 30, 60, 90, and 120 min. Two days later, the mice were subjected to 2 h fasting for the ipITT, and the glycemia was measured before (time 0) and 4, 8, 12, 16, 20, 30, and 60 min after the i.p. administration of 1 U/kg insulin. The KITT (glucose disappearance rate) was calculated as previously described (Akinmokun et al., 1992).

### Physiological Measurements

For indirect calorimetry, the mice were allowed 24h for acclimation to the apparatus 2 days after the ipITT. The mice remained at rest during a 12 h light/dark cycle. O_2_ and CO_2_ were measured by an Oxylet system (Pan Lab/Harvard Apparatus). The RQ was calculated from this data.

### Plasma Insulin and C-Peptide Measurements

Mouse insulin and C-peptide Elisa Kits (Darmstadt, Germany, cat. #EZRMI-13K and #EZRMCP2-21K) were used to measure plasma insulin and C-peptide. The plasma samples were obtained by centrifugation of blood samples at 11000 rpm, 15 min, 4°C. The assays were performed as indicated by the kit protocol. The blood samples for insulin and C-peptide measurements were collected in fed and fasted states at the end of the treatment.

### Insulin Degrading Enzyme (IDE) Activity

Liver IDE activity measurement was performed using the SensoLyte 520 IDE Activity Assay Kit (AnaSpec, Fremont, CA, United States, cat. AS-72231) following the manufacturer’s instructions. The total IDE activity was calculated as previously described (Kurauti et al., 2016) and normalized per μg of total protein, which was determined using Bradford reagent. The kinetic concentration of 5-FAM was also normalized per μg of total protein.

### Western Blot Analysis

Liver samples were collected and homogenized. After centrifugation at 12000 rpm for 30 min, 4°C, the protein was determined using Bradford reagent. For SDS (sodium dodecyl sulfate) polyacrylamide gel electrophoresis, all samples were treated with a Laemmli buffer containing dithiothreitol. After heating to 100°C for 5 min, proteins were separated by electrophoresis in a 10% polyacrylamide gel. The transfer to nitrocellulose membranes was performed in a Trans Blot transfer for 2 h in 100 V, with a tris/glycine buffer. After, the membranes were blocked with 5% BSA for 1 h and were then incubated with polyclonal antibodies against IDE (Abcam, cat. Ab32216). Tubulin (Sigma Aldrich, cat. 6074) was used as a control. Visualization of specific protein bands was performed by incubating the membranes with appropriate secondary antibodies. Protein bands were visualized using the Amersham Imager 600 (GE Healthcare Life Sciences, Buckinghamshire, United Kingdom), which detected chemiluminescence. The band intensities were quantified with ImageJ software (National Institutes of Health, Bethesda, MD, United States). Full scans of the entire original nitrocellulose membranes are available on Supplementary information (Supplementary Figures 1–4).

### Pancreas Morphometry and Immunohistochemistry

Pancreas samples from four animals from each group were fixed in 10% formalin, embedded in Paraplast (Sigma Aldrich, St. Louis, MO, United States), sectioned into slices of 5 μm and adhered to individual silanized glasses. The first and the 50th sections were immunoperoxidase-stained for insulin to quantify the distribution of pancreatic beta-cells. After the removal of the Paraplast, the sections were rehydrated and washed with 0.5 M Tris–buffered saline (TBS, pH 7.4) and treated with 0.1 M sodium citrate buffer (pH 6.0) at 100°C for antigen retrieval. The sections were washed again with TBS and blocked against endogenous peroxidase activity with 3% H_2_O_2._ Sections were washed with TBS and then incubated for 1 h with TBS and 3% BSA followed by primary antibody incubation overnight at 4°C. The antibody used was polyclonal guinea pig anti-insulin (Dako North America, Carpinteria, CA, United States, ref. A0564) diluted at 1:90 in TBS with 3% BSA. Subsequently, sections were incubated in the presence of a secondary antibody for 1 h. The antibody used was anti-guinea pig IgG, diluted at 1:200. The positive insulin cells were detected with DAB solution (0.1% DAB and 0.02% H_2_O_2_ in TBS). Finally, the sections were rapidly stained with Harris’ hematoxylin and mounted for microscopic observation. All islets present in the sections were covered systematically by capturing images with a digital camera coupled to a microscope (Olympus DP71; Olympus BX60, Japan). Pancreatic islets and beta-cells were measured using ImageJ software (National Institutes of Health, Bethesda, MD, United States). The pancreas weight was obtained immediately after the euthanasia. After immunohistochemistry, the measurement of the circumference of each islet was denominated as the total area of the islet. Islet/pancreas section ratio was presented as the total amount of islets in each section analyzed. The islet and beta-cell mass were calculated by multiplying the total islet area and beta-cell area by the pancreas weight (mg) (Lubaczeuski et al., 2015), and the beta cell/islet ratio was obtained by the total beta-cell area divided by the respective islet area.

### Statistical Analysis

The data are presented as the means ± standard errors mean (SEM) for 4–21 mice. To evaluate data normality, we applied the Shapiro-Wilk test. When normal, we used the parametric Student’s *t*-test; otherwise, the non-parametric Mann-Whitney test was adopted. The difference between groups was considered statistically significant if *P* ≤ 0.05.

## Results

### Streptozotocin Induced Type 1 Diabetes

**FIGURE 2:**
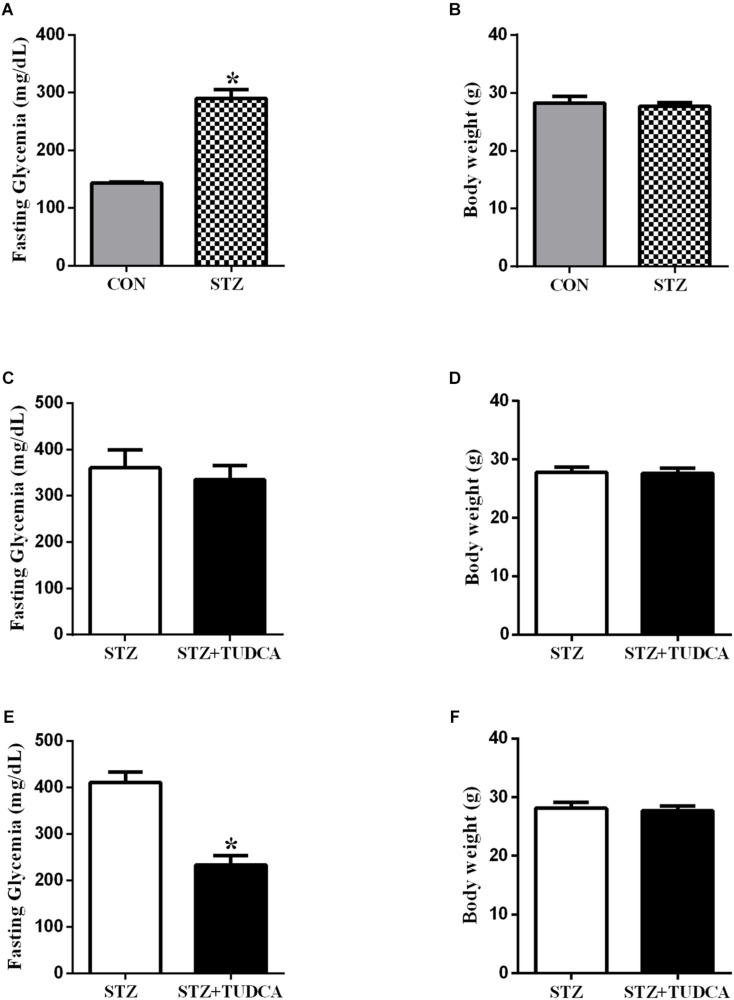
TUDCA treatment improves fasting glycemia from early-stage T1D mice. Fasting glycemia of CON (gray bar, *n* = 6) and STZ (grid bar, *n* = 21) **(A)**. Body weight of CON (gray bar, *n* = 6) and STZ (grid bar, *n* = 21) **(B)** at the end of the streptozotocin administration. Fasting glycemia of STZ (white bar, *n* = 10) and STZ + 7 days of TUDCA treatment (black bar, *n* = 11) **(C)**. Body weight of STZ (white bar, *n* = 10) and STZ + 7 days of TUDCA treatment (black bar, *n* = 11) **(D)**. Fasting glycemia of STZ (white bar, *n* = 10) and STZ + 15 days of TUDCA treatment (black bar, *n* = 11) **(E)**. Body weight of STZ (white bar, *n* = 10) and STZ + 15 days of TUDCA treatment (black bar, *n* = 11) **(F)**. Data are the mean ± SEM. *P ≤ 0.05 (Student’s *t*-test or Mann-Whitney test).

First, we characterized the early-stage T1D mice using fasting glucose measurement, and, as expected, the STZ mice presented hyperglycemia compared to the CON group (Figure 2A). However, no differences in body weight were noticed between both groups (Figure 2B). Because the experimental model for T1D worked, the subsequent experiments were done on STZ mice. Also, the pancreatic beta-cell mass has been addressed as well as insulin levels to confirm early-stage T1D, with reduction of beta-cell mass and insulin levels in STZ-treated mice (Supplementary Figures 6, 7).

### TUDCA Improved Fasting Glycemia in STZ Mice

In the first week of treatment, fasting glycemia (Figure 2C) and body weight (Figure 2D) in the STZ+TUDCA group were similar to the STZ group. However, at the end of the second week of treatment, fasting glycemia in the STZ+TUDCA mice was reduced by approximately 43% (Figure 2E), compared to the STZ mice. Body weight remained similar in both groups (Figure 2F). In addition, there was no difference between the groups in gastrocnemius muscle and perigonadal fat pad weight (Table 1).

**TABLE 1:** Final characterization of STZ and STZ+TUDCA mice (Student’s *t*-test was used for body weight; Mann-Whitney test was adopted for the other parameters, *P* ≤ 0.05).

	STZ	STZ+TUDCA
Body weight (g)	27.35 ± 1.08	27.85 ± 0.69
Perigonadal fat pad weight (g)	0.34 ± 0.03	0.45 ± 0.09
Gastrocnemius muscle weight (g)	0.51 ± 0.03	0.53 ± 0.02

*Data are the mean ± SEM (*n* = 6).*

### TUDCA Improved Glucose Tolerance but Did Not Alter Insulin Sensitivity in STZ Mice

**FIGURE 3:**
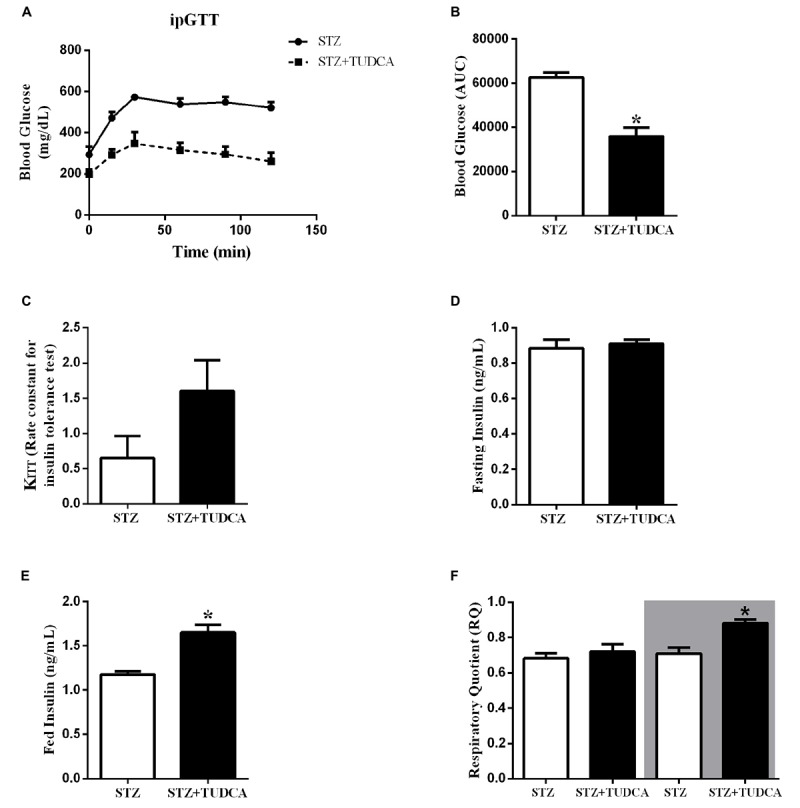
TUDCA treatment improves glucose tolerance, increases insulinemia in a fed state and improves metabolic flexibility in early-stage T1D mice. Blood glucose of STZ (white bar, *n* = 8) and STZ+TUDCA (black bar, *n* = 8) during ipGTT **(A)**. Area under the curve (AUC) of total blood glucose concentration of STZ (white bar, *n* = 8) and STZ+TUDCA (black bar, *n* = 8) during ipGTT **(B)**. Glucose disappearance rate during ipITT (KITT) of STZ (white bar, *n* = 4) and STZ+TUDCA (black bar, *n* = 6) **(C)**. Plasma insulin of STZ (white bar, *n* = 8) and STZ+TUDCA (black bar, *n* = 10) in fasting **(D)** and fed **(E)** states. Respiratory coefficient (RQ) **(F)** was determined by indirect calorimetry, during a 12-hour light/dark cycle. RQ of STZ (white bar, *n* = 4) and STZ+TUDCA (black bar, *n* = 4) during the light period. In addition, RQ of STZ (white bar with gray background, *n* = 4) and STZ+TUDCA (black bar with gray background, *n* = 4) during the dark period. Data are the mean ± SEM. *P ≤ 0.05 (Student’s *t*-test or Mann-Whitney test).

To investigate the effects of TUDCA on glucose homeostasis, we performed intraperitoneal glucose and insulin tolerance tests (ipGTT and ipITT). After glucose administration, both groups had a maximal glucose peak at 15–30 min (Figure 3A). However, the STZ+TUDCA mice presented an improved glucose tolerance (Figure 3A), as determined by the lower AUC of blood glucose during ipGTT (Figure 3B). As expected, there was no difference between the groups regarding insulin sensitivity, as determined by the glucose disappearance rate (KITT) (Figure 3C). Finally, we observed that plasma insulin levels in a fed, but not fasted, state was significantly higher in the STZ+ TUDCA group (Figures 3D,E).

### TUDCA Reduced Insulin Clearance, and IDE Activity, but Not IDE Expression, in the Liver of STZ Mice

**FIGURE 4:**
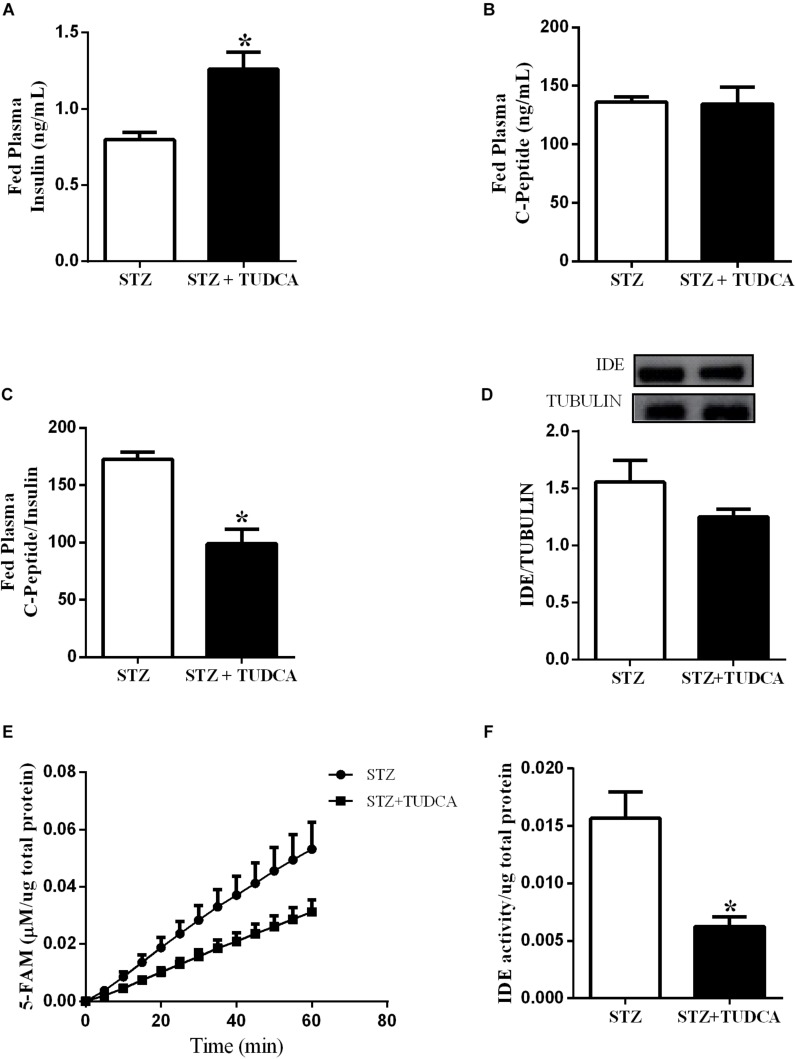
TUDCA reduces insulin clearance and Insulin Degrading Enzyme (IDE) activity, but not IDE expression, in the liver of STZ mice. Plasma insulin of STZ (white bar, *n* = 7) and STZ+TUDCA (black bar, *n* = 7) in fed state **(A)**. Plasma C-peptide of STZ (white bar, *n* = 7) and STZ+TUDCA (black bar, *n* = 7) in fed state **(B)**. C-peptide/insulin ratio of STZ (white bar, *n* = 7) and STZ+TUDCA (black bar, *n* = 7) in fed state **(C)**. Protein expression of IDE in the liver and its representative immunoblotting images of STZ (white bar, *n* = 8) and STZ+TUDCA (black bar, *n* = 8) **(D)**. Kinetic of IDE activity assay **(E)** and total IDE activity **(F)** in the liver of STZ (white bar, *n* = 4) and STZ+TUDCA (black bar, *n* = 5) mice. Fluorescent intensity at Ex/Em = 490/520 nm was recorded every 5 min for 60 min. 5-FAM concentration was calculated using a standard curve and normalized per μg of total protein. Data are the mean ± SEM. *P ≤ 0.05 (Student’s *t*-test or Mann-Whitney test).

Plasma insulin concentration is controlled by insulin secretion and clearance. In this case, elevated plasma insulin concentration in the STZ+TUDCA mice in a fed state could be due to a reduced insulin clearance. Therefore, we investigated the insulin clearance by the calculation of C-peptide/insulin ratio after a glucose challenge. As expected, the plasma insulin level was higher in the STZ+TUDCA, compared to STZ mice (Figure 4A). However, the plasma C-peptide was not different between groups (Figure 4B) suggesting a reduction in insulin clearance, confirmed by the reduction of C-peptide/insulin ratio in the STZ+TUDCA, compared to STZ mice (Figure 4C). The enzyme responsible for insulin clearance is IDE and the major site of insulin degradation is the liver. Thus, we evaluated IDE expression and activity in this organ. TUDCA treatment did not alter IDE expression (Figure 4D), however, IDE activity in the STZ+TUDCA was reduced, compared to STZ mice (Figures 4E,F), which explained, at least in part, the elevated plasma insulin concentration in this group.

### TUDCA Treatment Increased Beta-Cell Number per Islet and Mass in STZ Mice

**FIGURE 5:**
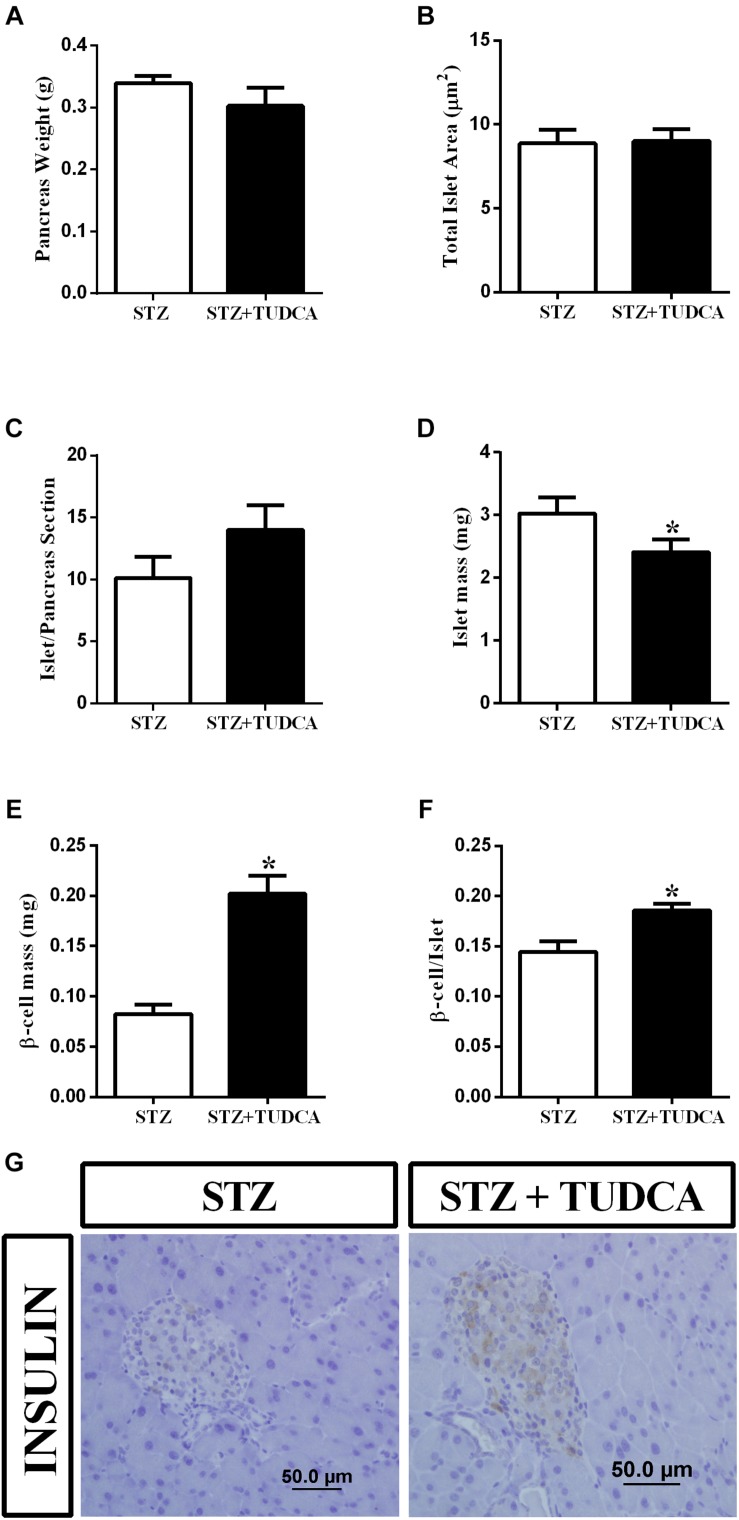
TUDCA treatment increases beta-cell mass and beta-cell number per islet in early-stage T1D mice. Pancreas weight (g) **(A)**; total islet area (μm${}^{2}$) **(B)**; islet/ pancreas section ratio **(C)**; islets mass (mg) **(D)**; beta-cell mass (mg) **(E)**; beta-cell/islet ratio **(F)** of STZ (white bar, *n* = 4) and STZ+TUDCA (black bar, *n* = 4) mice. Representative images of pancreas sections stained for insulin **(G)**. Data are the mean ± SEM. *P ≤ 0.05 (Student’s *t*-test or Mann-Whitney test).

We also investigated the effect of TUDCA treatment on beta-cell and islet morphology. We observed that pancreas weight (Figure 5A), total islet area (Figure 5B) and islet/pancreas section ratio (Figure 5C) remained similar in both groups. However, islet mass (Figure 5D) was reduced by 10% in the STZ+TUDCA group. On the other hand, beta-cell mass (Figure 5E) and the beta-cell number per islet (Figure 5F) increased by 144 and 28%, respectively, in the STZ+TUDCA mice compared with the STZ mice. Representative figures of the histological pancreatic sections, stained for insulin, are shown in Figure 5G.

### TUDCA Improved Metabolic Flexibility in STZ Mice

Tauroursodeoxycholic acid treatment increased insulinemia and reduced glycemia in the STZ mice. Thus, we also investigated whether these results may alter the metabolic flexibility of these mice by calculating the RQ. The RQ was calculated by measuring the amount of carbon dioxide (CO_2_) produced in comparison to the amount of oxygen (O_2_) used, and it is possible to predict which substrate is being oxidized and used as a fuel. As expected, during the light period, both groups presented a RQ ∼0.7, indicating a predominant use of fatty acid, because they were at rest and fasting (Figure 3F). On the other hand, during the dark period, when they were more active and fed, the RQ of the STZ group remained ∼0.7, indicating that they maintained a high rate of fatty acid oxidation; whereas the STZ+TUDCA mice presented an RQ ∼1.0, suggesting that they used predominantly carbohydrate oxidation (Figure 3F). Therefore, increased plasma insulin concentration in the STZ+TUDCA group may increase glucose uptake and oxidation, contributing to the reduced glycemia.

## Discussion

Type 1 diabetes is a highly prevalent disease characterized by hyperglycemia due to pancreatic beta-cell loss induced by an autoimmune attack (Chiang et al., 2014), and if not well controlled, it might lead to retinopathy, nerve damage, kidney and cardiovascular disease, and death (Atkinson et al., 2014, 2017). Daily exogenous insulin administration is the main therapeutic strategy for T1D and has been applied over the past decades. However, insulin therapy may induce important side effects, such as hypoglycemia, weight gain and cancer (Dewitt and Hirsch, 2003; Bergenstal et al., 2010; Wu et al., 2015). Thus, there is a search for molecules that could improve glucose control in T1D and contribute to the reduction of insulin dosing, and eventually the deleterious secondary effects mentioned above. In this context, the bile acid TUDCA has emerged as an important candidate due to its known benefits for type 2 diabetic patients, improving the insulin sensitivity in their peripheral tissues (Ozcan et al., 2006; Kars et al., 2010). In addition, TUDCA increases insulin secretion in isolated pancreatic islet from healthy mice (Vettorazzi et al., 2016), and prevents T1D development in NOD mice, treated with the compound during the pre-diabetic stage (Engin et al., 2013). Since a clinical trial with diabetic patients receiving TUDCA treatment (Goland and colleagues, Columbia University), is ongoing, we believe that understanding the mechanism of action of this bile acid upon glycemic control is important. Taking this into account, we treated early-stage T1D mice with TUDCA after the induction of the disease by multiple low doses of streptozotocin. The multiple low doses of streptozotocin approach causes partial pancreatic islet damage, activating an inflammatory process that further induces loss of beta-cell activity, ultimately resulting in a mild insulin deficiency and hyperglycemia, without insulin resistance which closely matches the early stage of T1D in humans (Lenzen, 2008; Furman, 2015). In our study, we aimed to verify the efficiency of TUDCA on a stage without complete loss of beta-cell, characterizing an early stage of the disease.

Twelve days after the last streptozotocin administration, the fasting blood glucose was almost 100% higher in the STZ group compared with the CON group. Also, STZ mice did not present alterations regarding insulin sensitivity, however, this result is a limitation of this study due to the low number of animals per group in this experiment. Moreover, the STZ-treated mice presented reduced insulinemia and beta-cell mass (Supplementary Figures 6, 7), consistent with previous streptozotocin multiple low doses T1D protocols (Furman, 2015; McKillop et al., 2016; Tekula et al., 2018; Khurana et al., 2018). After streptozotocin administration, we divided the mice into two groups; one received TUDCA (300 mg/kg) daily through i.p. injections for 24 days. On the 7th day, the glycemia was not different between the T1D groups. However, after 15 days of treatment, the fasting glycemia of the STZ+TUDCA group was significantly reduced compared with the STZ mice, without any changes in the body weight. The efficiency of TUDCA in reducing glycemia was confirmed by an ipGTT (Figure 3A), and it is possible that this improvement in glucose tolerance was due to the higher plasma insulin levels observed in these mice.

Insulinemia depends on a balance between insulin secretion and degradation. In healthy subjects, the liver is the main site for insulin degradation, which occurs through the involvement of IDE (Duckworth et al., 1998). IDE malfunction has been identified in obesity and type 2 diabetic models (Karamohamed et al., 2003; Erdmann et al., 2009). However, the possible role of hepatic IDE modulation and its impact upon insulin clearance in early or advanced T1D mice treated with TUDCA has not been investigated yet.

Here, we measured the IDE protein content in the liver and did not find differences between the T1D groups. However, we observed a decrease in the liver IDE activity in the STZ+TUDCA mice compared to the STZ mice, suggesting that the lower IDE activity in the TUDCA treated mice might contribute to reduced insulin degradation, justifying the higher insulinemia in the STZ+TUDCA mice. Indeed, reduced hepatic IDE activity, observed in the STZ+TUDCA mice, may explain the reduction in the insulin clearance in this group. It is known that insulin clearance is increased in the streptozotocin diabetic rodent model (Philippe et al., 1981), and our findings suggest TUDCA treatment as an important strategy to improve the impaired insulin clearance in the T1D patients. These results negate previous data from our group, which showed that TUDCA increased the insulin clearance by increasing IDE expression. However, this study was performed in hyperinsulinemic mice fed with a high fat diet (Vettorazzi et al., 2017), suggesting that the TUDCA-induced IDE modulation is dependent on the insulinemic state.

The effects of TUDCA on the liver do not exclude the possibility of the involvement of pancreatic islets in this context. Thus, we evaluated the histology of pancreas from early-stage T1D mice and observed that the STZ+TUDCA mice presented increased beta-cell mass and beta-cell number per islet, suggesting that TUDCA may be able to partially recover beta-cell mass, which, together with the reduction in IDE activity and insulin clearance, results in an increased insulinemia and, consequently, improvement of glucose homeostasis in T1D mice. The reduction in ER stress and inflammation could be the molecular mechanism by which TUDCA improves pancreatic islet homeostasis and beta-cell mass in this study. In fact, previous studies have shown the chaperone function of TUDCA in pancreatic islets and beta-cells (Lee et al., 2010; Cadavez et al., 2014). Also, Engin et al. (2013) shown that TUDCA treatment, during the pre-diabetic stage, reduces T1D incidence in RIP-LCMV-GP (rat insulin promoter–lymphocytic choriomeningitis virus–glycoprotein), keeping the pancreatic islet largely intact, despite the presence of inflammatory cells, and reduces beta-cell apoptosis by reducing ER stress in an ATF6-dependent pathway.

Paradoxically, the STZ+TUDCA mice showed reduced islet mass compared to the STZ mice. We speculate that this feature might be a result of reduced immune cell infiltration into the islets, since it was demonstrated that TUDCA prevents insulitis in NOD mice in the pre-diabetic state (Engin et al., 2013). Moreover, TUDCA treatment protects human pancreatic islet, exposed to a combination of pro-inflammatory cytokines (IL-1β, TNF-α and IFN-γ), reducing apoptosis induced by these cytokines (Ocaña et al., 2017).

The T1D patients display metabolic inflexibility due to their incapacity to adjust uptake of macronutrients according to their metabolic needs (Kelley and Mandarino, 2000). Healthy mice preferentially oxidize glucose during the dark cycle (fed state) and fatty acids in the light cycle (fasted state) (Randle, 1986). Here, we demonstrated that TUDCA treatment was able to improve the energetic metabolism in T1D mice, probably due to their insulinemia recovery.

Taken together, we concluded that TUDCA treatment increased insulinemia in the STZ-induced diabetic mice in an early stage of the disease through two distinguished mechanisms: increasing pancreatic beta-cell mass and decreasing IDE activity in the liver and consequently reducing insulin clearance. Both alterations contribute to the improvement of glycemic control and metabolic flexibility in these mice. These findings suggest that TUDCA treatment is a potential strategy to counteract glucose homeostasis disturbance in early-stage type 1 diabetic patients.

## Ethics Statement

This study was carried out in accordance with the recommendations of Conselho Nacional de Controle de Experimentação Animal (CONCEA),Comissão de Ética no Uso de Animais da Universidade Estadual de Campinas - CEUA/UNICAMP. The protocol was approved by the Comissão de Ética no Uso de Animais da Universidade Estadual de Campinas - CEUA/UNICAMP.

## Author Contributions

GB, JCJ, and JV contributed to research design. GB, JCJ, JV, GS, MK, CS, and MB conducted the experiments and acquired data. AB and EC provided all reagents. GB, JCJ, JV, GS, and MK contributed to data analysis and interpretation. GB and JCJ wrote the manuscript. AB and SB revised the manuscript. All authors reviewed and approved the final version of the manuscript.

## Conflict of Interest Statement

The authors declare that the research was conducted in the absence of any commercial or financial relationships that could be construed as a potential conflict of interest.

## Abbreviations

AKT: Protein kinase B
ATF6: Activating transcription factor 6
AUC: Area under the curve
BSA: Bovine Serum Albumin
CON: Control
DAB: Diaminobenzidine
ER: Endoplasmic Reticulum
IDE: Insulin degrading enzyme
NOD: Non-obese diabetic
PBS: Phosphate buffered saline
PI3K: Phosphoinositide 3-kinase
RIP-LCMV-GP: Rat insulin promoter–lymphocytic choriomeningitis virus–glycoprotein
RQ: Respiratory quotient
S1PR2: Sphingosine-1-phosphate receptor 2
SDS: Sodium dodecyl sulfate
STZ: Streptozotocin
T1D: Type 1 diabetes
TBS: Tris–buffered saline
TUDCA: Tauroursodeoxycholic acid
UDCA: Ursodeoxycholic acid.
