# Supplementary material for: The Bile Acid TUDCA Improves Beta-Cell Mass and Reduces Insulin Degradation in Mice With Early-Stage of Type-1 Diabetes
Source: Front Physiol. 2019 May 15;10:561. doi: 10.3389/fphys.2019.00561 (PMC6529580; doi:10.3389/fphys.2019.00561)
Supplement: Supplementary file 1 [file Data_Sheet_1.pdf]

Supplementary Material

The bile acid TUDCA improves beta-cell mass and reduces insulin degradation in mice with early-stage of Type-1 diabetes

Gabriela Alves Bronczek[1,2#], Jean Franciesco Vettorazzi[2#], Gabriela Moreira Soares[2], Mirian Ayumi Kurauti[2], Cristiane Santos[2], Maressa Fernandes Bonfim[2], Everardo Magalhães Carneiro[2], Sandra Lucinei Balbo[1], Antonio Carlos Boschero[2], José Maria Costa Júnior[2*]

*** Correspondence:** Dr José Maria Costa Júnior: josefisioexer@gmail.com

1 Supplementary Figures

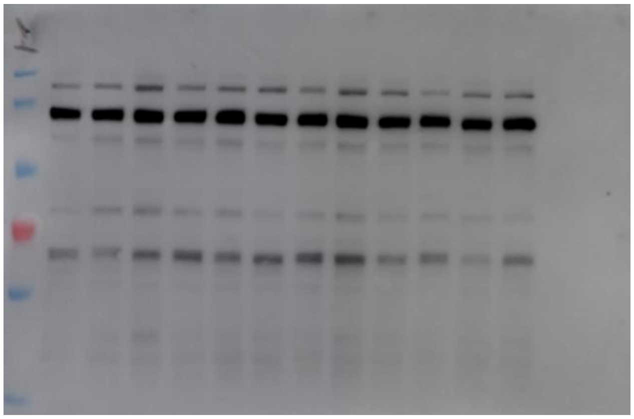

Supplementary Figure 1. Full scan of the entire original gel (1) incubated with IDE (insulin degrading enzyme) antibody. The samples were transferred to nitrocellulose membranes in this sequence: CON, STZ and STZ+TUDCA. However, the CON group was excluded from the official results.

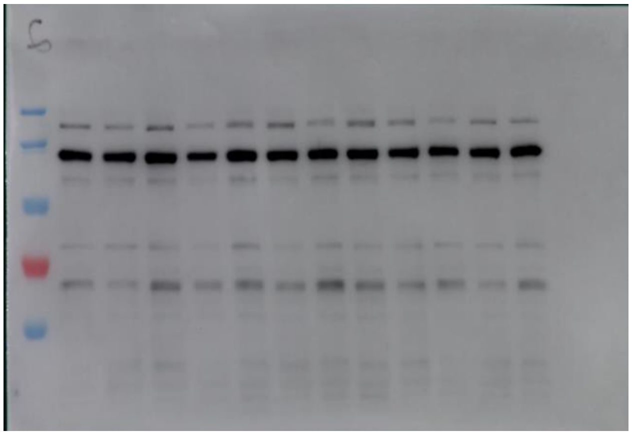

Supplementary Figure 2. Full scan of the entire original gel (2) incubated with IDE (insulin degrading enzyme) antibody. The samples were transferred to nitrocellulose membranes in this

sequence: CON, STZ and STZ+TUDCA. However, the CON group was excluded from the official results.

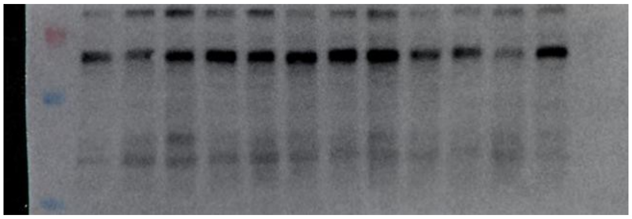

Supplementary Figure 3. Full scan of the entire original gel (1) incubated with α-Tubulin antibody. The samples were transferred to nitrocellulose membranes in this sequence: CON, STZ and STZ+TUDCA. However, the CON group was excluded from the official results.

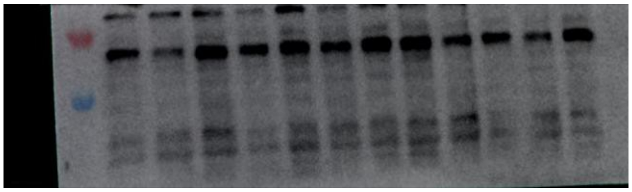

Supplementary Figure 4. Full scan of the entire original gel (2) incubated with α-Tubulin antibody. The samples were transferred to nitrocellulose membranes in this sequence: CON, STZ and STZ+TUDCA. However, the CON group was excluded from the official results.

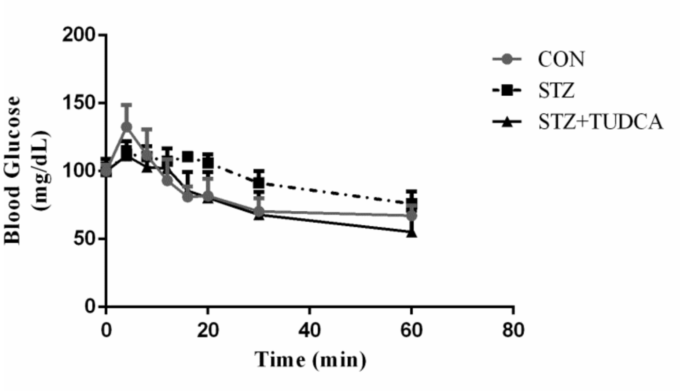

Supplementary Figure 5. Blood glucose during ipITT of CON (n=5), STZ (n=4) and STZ+TUDCA (n=6). Data are the mean ± SEM.

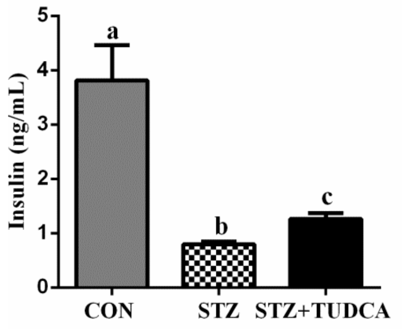

Supplementary Figure 6. Plasma insulin of CON (n=5), STZ (n=7) and STZ+TUDCA (n=7) in fed state. Data are the mean ± SEM. Different letters represent P ≤ 0.05 (ANOVA).

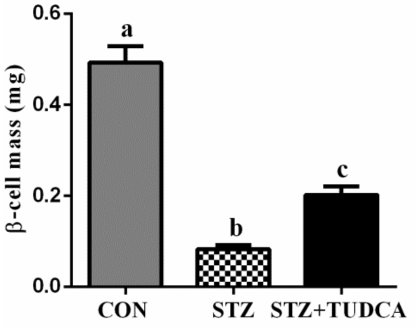

Supplementary Figure 7. Beta-cell mass (mg) of CON (n=4), STZ (n=4) and STZ+TUDCA (n=4). Data are the mean ± SEM. Different letters represent P ≤ 0.05 (ANOVA).
